# COVID-Dynamic: A large-scale longitudinal study of socioemotional and behavioral change across the pandemic

**DOI:** 10.1038/s41597-022-01901-6

**Published:** 2023-02-03

**Authors:** Tessa Rusch, Yanting Han, Dehua Liang, Amber R. Hopkins, Caroline V. Lawrence, Uri Maoz, Lynn K. Paul, Damian A. Stanley, the COVID-Dynamic Team, Ralph Adolphs, R. Michael Alvarez, Isabella Camplisson, Laura Harrison, Denise Hien, Tian Lan, Chujusn Lin, Teresa Lopez-Castro, Marie-Christine Nizzic, Allison Rabkin Golden, Iman Wahle, Gideon Yaffe

**Affiliations:** 1 Division of Humanities and Social Sciences, California Institute of Technology, Pasadena, CA USA; 2 Division of Biology and Biological Engineering, California Institute of Technology, Pasadena, CA USA; 3 Institute for Interdisciplinary Brain and Behavioral Sciences, Chapman University, Orange, CA USA; 4 Yale Law School, New Haven, CT USA; 5 Derner School of Psychology, Adelphi University, 1 South Avenue, Garden City, NY 11530 USA; 6 Center of Alcohol & Substance Use Studies, Graduate School of Applied and Professional Psychology, Rutgers University-New Brunswick, Piscataway, NJ USA; 7 Department of Psychology and Brain Sciences, Dartmouth College, Hanover, NH USA; 8 Psychology Department, The City College of New York, NY, USA; 9 Arts and Science Faculty, Dartmouth College, Hanover, NH USA

**Keywords:** Human behaviour, Decision making, Risk factors, Society

## Abstract

The COVID-19 pandemic has caused enormous societal upheaval globally. In the US, beyond the devastating toll on life and health, it triggered an economic shock unseen since the great depression and laid bare preexisting societal inequities. The full impacts of these personal, social, economic, and public-health challenges will not be known for years. To minimize societal costs and ensure future preparedness, it is critical to record the psychological and social experiences of individuals during such periods of high societal volatility. Here, we introduce, describe, and assess the COVID-Dynamic dataset, a within-participant longitudinal study conducted from April 2020 through January 2021, that captures the COVID-19 pandemic experiences of >1000 US residents. Each of 16 timepoints combines standard psychological assessments with novel surveys of emotion, social/political/moral attitudes, COVID-19-related behaviors, tasks assessing implicit attitudes and social decision-making, and external data to contextualize participants’ responses. This dataset is a resource for researchers interested in COVID-19-specific questions *and* basic psychological phenomena, as well as clinicians and policy-makers looking to mitigate the effects of future calamities.

## Abstract (summary)

**Table:** 

Measurement(s)	Mental and Physical Health, Behavior, Personality, Emotion, Racial and Political Attitudes (Implicit and Explicit), Social and Moral Decision-Making, Demographics
Technology Type(s)	Questionnaires and Choice Tasks
Factor Type(s)	none
Sample Characteristic - Organism	Adult Humans
Sample Characteristic - Environment	Online Survey
Sample Characteristic - Location	United States of America

## Background & Summary

**Fig. 1:**
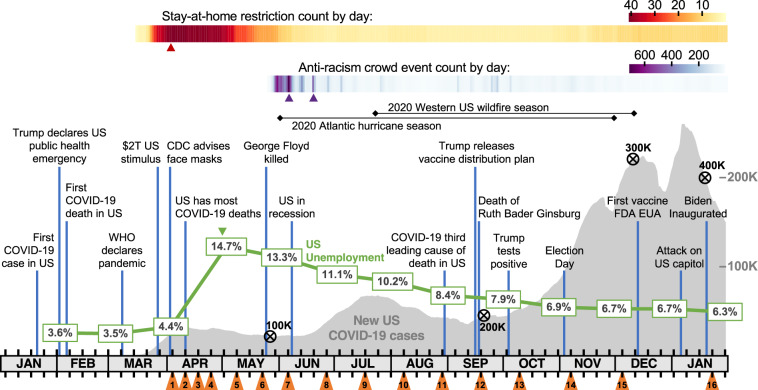
Timeline of Real-World Events and COVID-Dynamic Wave Administrations. Visualization of the COVID-Dynamic data collection schedule in the context of the events of January 2020 to January 2021. Orange triangles denote each wave administration (black tick marks depict weekly intervals). The gray curve indicates the daily 7-day average of new, confirmed COVID-19 cases in the U.S.^49^, black encircled X’s on top of the curve mark grim U.S. COVID-19-related death milestones (100,000 to 400,000 dead). The green line shows the monthly US unemployment rate^2^. The upper gradient (yellow-red) indicates the daily count of states with active stay-at-home restrictions (peak = 41)^3^. The lower gradient (blue-purple) shows the daily count of U.S. anti-racism crowd events^58^. Colored triangles below the gradients indicate local maxima for the various measures. All these external data (aligned to our data collection) are included in the dataset. Events of interest are indicated with vertical blue lines.

The COVID-19 pandemic has been a global catastrophe. In the United States, as of early 2021, (Fig. 1) the pandemic had triggered tremendous societal upheaval with over 400,000 COVID-19-related deaths, an economic downturn resembling the Great Depression^1,2^, and prolonged social-isolation, the detrimental effects of which will take years to understand. In mid-March of 2020, it was apparent that the societal effects of COVID-19 would be extreme, idiosyncratic, and highly dynamic, providing a unique opportunity to examine the human psychological response to crisis. To capture this moment, we developed the COVID-Dynamic longitudinal study (www.coviddynamic.caltech.edu): a set of survey- and task- based measures, designed to characterize and quantify the dynamics of psychological, emotional, moral, attitudinal, and behavioral change over the COVID-19 pandemic (see *Methods – Measures*, https://osf.io/sb6qx for pre-registration, https://osf.io/8bg9f/ for a continuously updated project-registry detailing the various ongoing sub-projects). We were motivated by the following goals:

1. Characterizing within-participant psychological dynamics: We sought to document the extent to which individual characteristics (e.g., states, traits, attitudes, etc.) change in the face of severe environmental stress. This topic has clear clinical relevance but also addresses basic questions about the stability of psychological characteristics.
2. Breadth of assessment: It was clear that the pandemic would impact many aspects of personal and social life. Therefore, we were inclusive in our selection of measures. We assessed mental and physical health, behavior, personality, emotion, racial and political attitudes (implicit and explicit), social and moral decision-making, along with demographic detail (see *Measures - COVID-Dynamic Test Battery*). To situate these data in context, we compiled a variety of public measures (e.g., COVID-19 and restriction-related metrics, unemployment numbers, anti-racism-protest counts).
3. Methodological diversity: Recent work^3^ highlights the importance of methodological diversity for psychological studies. We therefore balanced standardized self-report measures (clinical and basic), with novel targeted surveys, implicit measures, consequential social decision-making tasks, free response measures, and behavioral reports. As such, the dataset provides a rare opportunity to explore within-subject differences related to measurement modality.
4. Sample representativeness: We tailored the survey recruitment to balance logistical feasibility with the goal of sample representativeness. Recruitment was limited to the US to optimize sampling density across geographic areas given funding constraints and researcher sensitivity to societal context. Recognizing that exact representativeness was unattainable, we conducted a thorough audit of biases in our sample.
5. Rigor, Transparency, and Open Science: We strongly value scientific rigor and transparency. Extensive data quality metrics obtained during and after data collection are provided alongside the raw data. All materials pertaining to the study are archived with the study preregistration (https://osf.io/sb6qx). Urgency and the unpredictable nature of what was to come rendered full preregistration of specific hypotheses impossible. Instead, the data were preprocessed using a central pipeline and access to requested data was granted to COVID-Dynamic Team members following submission and team approval of specific analyses. To maintain future transparency for the public dataset, it is licensed under Creative Commons By Attribution (CC BY 4.0) and all users of the data are encouraged to submit a project description (https://osf.io/8bg9f/) that will be added to the public project repository.

The resulting COVID-Dynamic dataset provides a unique inventory of the psychological, emotional, moral, attitudinal, and behavioral changes, as well as the personal experiences of a large (1000+) cohort of U.S. residents from all 50 states during this unparalleled time. Many other studies have queried the psychological effects of the pandemic but to our knowledge, none have captured a comparable breadth, sample size, testing frequency, and duration as the COVID-Dynamic study (see https://adolphslab.github.io/CVD-DYN_datarelease/).

The pandemic was not the sole stressor of 2020 in the United States. Racial and ethnic disparities in pandemic-related health and economic outcomes^4,5^, the horrific killing of George Floyd^6^, and increased Anti-Asian harassment^7^ highlighted and exacerbated existing societal fissures. Concurrently, political polarization escalated during the 2020 presidential election. From its inception the COVID-Dynamic study cast a wide net, assessing political beliefs, racial attitudes, social and moral norms, and social biases. As such, the COVID-Dynamic dataset is uniquely positioned to tackle myriad exciting and unanswered questions across many distinct psychological and social domains.

In the face of the extraordinary events of 2020, it is crucial that we, as a society, document the objective *and subjective* experiences of those affected—both to understand what happened and to plan ahead. The insights gained from the COVID-Dynamic dataset provide a knowledge base upon which to form policy and organize action. Hence, in addition to its contribution to understanding the effects of the COVID-19 pandemic and basic psychological science, this dataset may also contribute to two clear goals of applied psychological research: mitigation of the impact of trauma and promotion of resilience in the face of adversity.

## Methods

### Procedures

The study procedures were preregistered on the Open Science Framework (OSF) website (https://osf.io/sb6qx) to ensure transparency. All procedures were reviewed and deemed exempt (apart from the ASSIST) by the Internal Review Board of the California Institute of Technology (protocol 20-0992). The ASSIST was deemed not exempt and was approved following review by the Internal Review Board of the California Institute of Technology under a separate protocol (protocol 20-1014).

Recruitment through Prolific.co began on April 4, 2020. In total 1830 participant places were made available on Prolific, and 1797 participants completed Wave 1. Prolific randomly invites participants from their pool of eligible participants. To ensure geographic and age representation in the sample better reflected that of the US population, we published the survey in eight batches stratified by US-state of residence and age (all places in each batch were filled unless indicated otherwise): all US states - ages 18–100 (30 invites), eastern US states - ages 18–100 (450 invites), eastern US states - ages 40–100 (150 invites), central US states - ages 18–100 (450 invites), central US states - ages 40–100 (150 invites), western US states - ages 18–100 (450 invites), western US states - ages 40–100 (110 out of 125 invites), and Hawaii and Alaska - ages 18–100 (2 out of 25 invites). 5 additional participants were not linked to any particular recruitment group. They completed the study via a direct survey link due to issues with administering the survey through Prolific. Each of the subsequent waves was posted on Prolific on a Saturday morning at 10am EDT and participants had 48 hours (until Monday at 10am EDT) to begin completing the wave’s survey. Each wave was designed to be completed in approximately 60 minutes and participants were allowed 140 minutes to complete it from the moment they accepted the task on Prolific.

Participants were paid at a standard rate of approximately $10/h. To reduce attrition, from Wave 5 onwards, 50 participants were randomly selected to receive a bonus of $10 multiplied by the proportion of total waves they had completed. Additionally, after each completed month, all participants were entered into a lottery for ten $50 bonuses. Odds were determined by their choices in the public goods game and the altruism task.

A researcher monitored the data collection in real time on Saturdays and Sundays between 10am EDT and midnight EDT and Mondays from 8am EDT until the closing of the wave. Monitors provided feedback to participants through Prolific’s anonymous messaging system and addressed any technical difficulties as they arose. From Wave 3 on, participants received two reminders to complete the wave during data collection (morning and afternoon on Sunday).

**Fig. 2:**
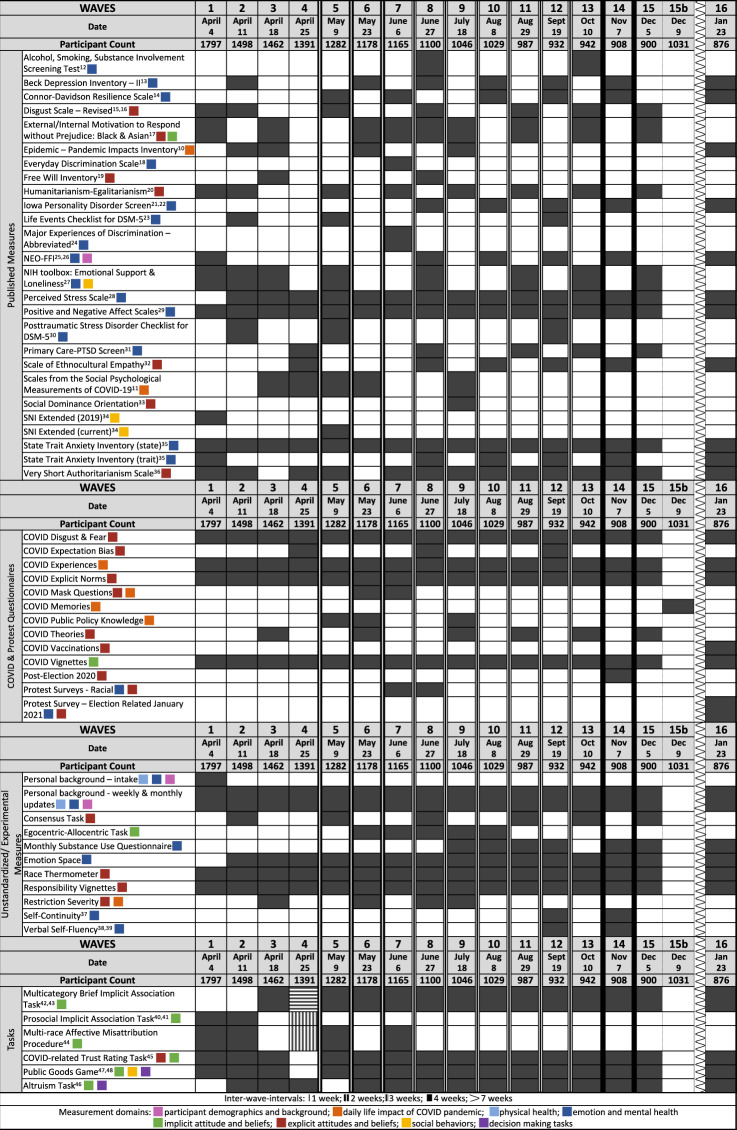
Administration Schedule of Questionnaires and Tasks. Visualization of the measure (rows) by wave (columns) administration schedule. Dates mark the day each wave was published. Measures are grouped into four categories: Published Measures, COVID & Protest Questionnaires, Unstandardized/ Experimental Measures, and Tasks. Filled cells (gray) indicate waves in which a particular measure was administered. Striped cells (gray/white) in the Task section at Wave 4 indicate that one half of the sample completed the Multicategory Brief Implicit Association Task (horizontal stripes) while the other half completed the Prosocial Implicit Association Task and the Multi-race Affective Misattribution Procedure (vertical stripes). Colored squares indicate researcher-assigned study domains. The line styles (e.g., single, double, triple, etc.) of the vertical lines between columns indicate the number of weeks between wave administrations.

Waves 1 through 4 were conducted weekly (April 4, 11, 18, and 25, 2020), followed by bi-weekly administration of Waves 5 through 7 (May 9, 23, and June 6, 2020). Subsequent waves were administered at intervals of 3 or 4 weeks, with 7 weeks between Waves 15 and 16 (see Fig. 2). Wave 15b was conducted in the week following Wave 15 (December 9) to collect one additional measure.

Testing was administered using a combination of the Qualtrics survey platform (questionnaires) and Pavlovia^8^ (tasks). While the specific content varied from wave to wave (see Fig. 2), the general sequence remained consistent. Participants first consented to participate and endorsed a level of commitment to the ongoing study. Next, they completed questionnaires regarding background, COVID experiences & mask use^9^, experiences related to protests and racial discrimination, and the Emotion-Space experimental measure. This was followed by the COVID Vignettes (implicit assessment of COVID norms), then the experimental tasks (in randomized order with the BIAT always last), the Responsibility Vignettes, and then various standardized and experimental questionnaires in randomized order (see *Measures* for details on questionnaires and tasks). Toward the end, the participants answered 3 questions about explicit norms related to COVID-19. In some waves, they also answered questions about how and where they believed COVID-19 originated. Finally, they were presented with the debriefing and a list of resources to aid with mental health issues, acute mental health crises, and food shortages. Participants were also provided with a PDF file containing a resource list at the beginning of the survey.

### Participants

#### Participant inclusion criteria

Participants were recruited and screened through Prolific (www.prolific.co) based on the following inclusion criteria: aged 18–100, English fluency, United States residency (50 states), Prolific approval rating of 98% or higher, and minimum of 5 Prolific studies completed. To avoid drawing heavily from one geographic location, recruitment from the 50 states was spread equally across 3 areas (US East Coast, Middle US, US West Coast). To counteract a bias towards younger adults that is often present in online studies, a portion of the recruitment specifically targeted individuals aged 40–100. In total, 1797 participants completed Wave 1 of the study.

#### Participant exclusion criteria

To ensure cost efficiency and data quality, for Waves 1–5 those participants who showed low levels of commitment and/or for whom data were deemed of poor quality, were excluded from continued recruitment following each wave. The exclusion criteria were as follows: unable to fully complete the experimental tasks in Wave 1 (27 participants), flagged on quality measures provided by Prolific (6 participants), missed >1 attention-check questions (15 participants); responded with less than 90% commitment to participation in future waves (94 participants; queried at beginning and end of each wave); and overall completion time below our minimum threshold (25, 30, or 35 minutes as determined for each wave through visual inspection of the study duration distribution and consensus among the COVID-Dynamic Team) (94 participants). Two participants requested to be excluded from further data collection after Wave 3. Application of these criteria resulted in the reduction of the study invitation list from 1797 to 1576 by the end of Wave 5. From Wave 5 on, it was decided by COVID-Dynamic Team consensus that no more participants would be excluded as the data from the remaining participants were likely to be of good quality. Nevertheless, data quality was assessed extensively in all waves and all data quality metrics are provided with the data (see *Technical Validation - Data Quality* for more details).

#### Core participants

**Fig. 3:**
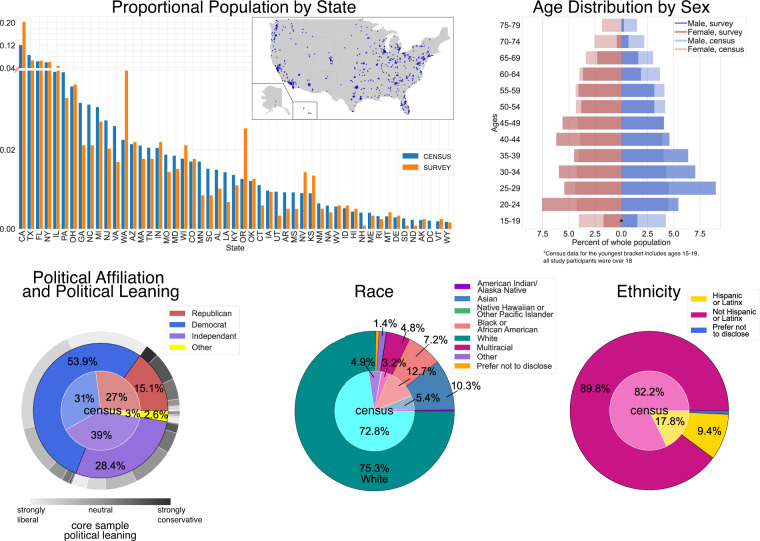
Core Sample Demographics Compared Against US Population. Core Sample (N = 1177) Demographics Compared Against US Population. Core sample demographics collected at Wave 1 compared to the overall US population (population by state, age, sex and race estimates were retrieved from the American Community Survey^61^, political affiliation estimates were retrieved from Gallup Historical Trends^62^). At the top left, the state-wise population proportion is shown sorted in descending order according to the 2018 American Community Survey (ACS) estimate (blue) together with the proportions of state representation in the core sample (orange). On the map inset, zip-code data (collected in Wave 8) are displayed which, due to attrition, comprise only 77% of the core sample. At the top right, the age distribution for males and females in the core sample is displayed along with that of the US population. Age brackets follow the ACS brackets (Note: all study participants were over 18). On the bottom row, pie charts of Political Affiliation and Political Leaning (left), Race (middle), and Ethnicity (right) are displayed for the US population (inner circle) and for our core sample (outer ring; except for bottom left panel, where the outermost ring designates political leaning in our core sample in grayscale). For readability, labels <1% were dropped from the pie charts.

To provide an overview of the data from the 16 waves presented here, a core sample was defined containing only those participants who had completed at least 50% (i.e., ≥8 waves) of the waves and who had not met any of the exclusion criteria described above. This core sample consisted of 1177 participants (51.2% female, median age = 39.4, age range = 18–77). Detailed demographic information for the core sample is presented in Fig. 3. Information about participants excluded from the core sample (i.e., the excluded and low-completion rate groups) is presented in the Technical Validation section. Sub-sample specific counts (invited, core, exclude and low-completion rate sample) across waves are shown in Fig. 5a. The data from all participants – i.e., core, excluded, and low-completion rate samples – are provided in the dataset. The sample membership of each participant is indicated in the “sample” variable (see Data Dictionary in *Data Records*).

### Measures

We set out to cast as wide a net as possible covering mental health, social behavior and decision-making, implicit and explicit attitudes and norms, substance abuse, public policy acceptance, and much more. Decisions about measure inclusion and wave-to-wave measure deployment were consensus-driven (capitalizing on expertise in the research domains represented by the COVID-Dynamic Team; https://coviddynamic.caltech.edu/investigators) with an emphasis on the aforementioned goals and areas of interest (see *Background & Summary*). In general, measures that assessed trait-like constructs were sampled less frequently than measures that assessed state-like constructs. In addition, measure deployment was constrained by duration (survey duration was kept close to an hour to make it predictable and not onerous for participants) and funding (balancing the high cost of each wave with the unknown duration of the pandemic). The full battery of questionnaires and tasks administered across the 16 waves is presented in Fig. 2 along with the schedule of administration. Detailed descriptions of each questionnaire and task are provided below. Additionally, all self-report questionnaires are available for download on OSF (https://osf.io/nhm2v/) allowing for detailed exploration of questionnaire items and structure.

#### COVID-Dynamic test battery

The self-report questionnaire battery included commonly used, published psychological assessment instruments and race-related surveys in addition to two publicly-available COVID19-specific instruments (Epidemic - Pandemic Impacts Inventory^10^, Scales from the Social Psychological Measurements of COVID-19^11^) (see *Measures - Published Measures*). We also administered a variety of self-report questionnaires created specifically for this study to characterize experiences (e.g., direct exposure to COVID-19, COVID-19 illness amongst family and friends, more general COVID-19-related changes to daily life) and attitudes (e.g., towards masking, government-mandated restrictions, the killing of George Floyd, etc.) related to COVID-19 and the Black Lives Matter (BLM) protests, as well as demographic surveys and experimental questionnaires related to new and ongoing research (see *Measures -* *Measures Created by COVID-Dynamic Team*). The specific self-report questionnaires are listed below.

1. Self-report questionnaires - Published Measures
  1.1. *Alcohol, Smoking, Substance Involvement Screening Test (ASSIST)*^12^ - a self-report, culturally-neutral instrument that measures degree of personal risk related to use of the following substances: tobacco products, alcohol, cannabis, cocaine, amphetamine-type stimulants (ATS), sedatives and sleeping pills (benzodiazepines), hallucinogens, inhalants, opioids, and ‘other’ drugs.
  1.2. *Beck Depression Inventory – II (BDI-II)*^13^ - a 12-item self-report questionnaire that examines depressive symptomatology over the prior 2 weeks. Total scores indicate general level of depression: none, mild, moderate, severe.
  1.3. *Connor-Davidson Resilience Scale - 10 Item (CD-RISC-10)*^14^ - a self-report index of resilience based on Likert-scale ratings of coping responses during the prior month.
  1.4. *Disgust Scale - Revised (DS-R)*^15,16^ - a 27-item self-report questionnaire that measures an individual’s sensitivity to disgust. It provides an index of core disgust (including food, animals, and body products), animal-reminder disgust (addressing death and organism violations) and contamination disgust (concerns about interpersonal transmission of materials).
  1.5. *External/Internal Motivation to Respond without Prejudice Scale (EMS*/*IMS): Black*^17^ *& Chinese (adapted)* - a brief self-report questionnaire, 10 items, that assesses the degree to which an individual’s attitudes and prejudice toward a designated racial group are motivated by internal values or external social pressures. The instrument was administered twice, once focused on Black people (the original scale) and once on Chinese people (adapted for this study).
  1.6. *Epidemic – Pandemic Impacts Inventory (EPII)*^10^ - a 92-item self-report instrument used to identify how the current pandemic has impacted various aspects of one’s life including: work/employment, education/training, home life, social activities, economic, emotional health and well-being, physical health, physical distancing/quarantine, infection history, and positive change.
  1.7. *Everyday Discrimination Scale*^18^ - a self-report measure of how frequently one experiences 9 types of discrimination in day-to-day life. For each type of discrimination that occurs “a few times a year” or more frequently, the individual indicates the main trait that is eliciting the discrimination.
  1.8. *Free Will Inventory (FWI)*^19^ - a self-report questionnaire in which individuals report how strongly they believe in free will, determinism, and dualism (15 items that produce a score for each of the 3 categories), as well as beliefs regarding relationships between free will, choice, responsibility, punishment, scientific prediction, and dualism (14 items).
  1.9. *Humanitarianism-Egalitarianism*^20^ - a 10-item self-report questionnaire that assesses the extent to which an individual adheres to ideals of equality, social justice, and concern for the well-being of others, i.e., humanitarian-egalitarian values.
  1.10. *Iowa Personality Disorder Screen (IPDS)*^21,22^ - an 11-item screening questionnaire used to identify the presence of personality disorder in nonclinical samples. A score of 2 or more on items 1 and 3–8 provides optimal sensitivity and specificity for screening.
  1.11. *Life Events Checklist for DSM-5 (LEC-5)*^23^ - a self-report questionnaire which asks about an individual’s lifetime experiences of 17 types of trauma.
  1.12. *Major Experiences of Discrimination – Abbreviated*^24^ - a self-report, lifetime frequency index of experiencing 6 types of major discrimination and the individual trait that elicited discrimination.
  1.13. *NEO Five-Factor Personality Inventory (NEO-FFI)*^25,26^ - a self-report questionnaire in which an individual rates how well each of the 60-items describes his/her behaviors, feelings, experiences and beliefs. In combination, these ratings indicate the degree to which the individual exhibits each of these 5 personality traits: extraversion, neuroticism, openness, conscientiousness, and agreeableness. Scoring algorithm and norms used for z-score conversion at http://www.uoregon.edu/~gsaucier/NEO-FFI%20subcomponent%20norms%20and%20scoring.htm.
  1.14. *NIH toolbox: Emotional Support & Loneliness*^27^ - brief self-report indices of how often an individual had access to emotional support (8 items) and felt lonely or alone in the past month (5 items). These measures are part of the NIH Toolbox (https://www.healthmeasures.net/explore-measurement-systems/nih-toolbox) and outcomes are reported as T-scores normed to the general population (based on 2010 US Census).
  1.15. *Perceived Stress Scale (PSS)*^28^ - a 10-item self-response questionnaire that measures the extent to which a participant perceives personal life events in the previous month as stressful.
  1.16. *Positive and Negative Affect Scales (PANAS)*^29^ - a 20-item self-report measure designed to assess the subjects’ current affective state.
  1.17. *Posttraumatic Stress Disorder Checklist for DSM-5 (PCL-5)*^23,30^ - a self-report questionnaire which asks about the degree to which 20 posttraumatic stress disorder symptoms were experienced during the prior month.
  1.18. *Primary Care-PTSD Screen (PC-PTSD)*^31^ - a brief self-report questionnaire in which participants are asked to report the presence of five post-traumatic stress disorder symptoms during the past month. Scores of 4 or 5 indicate probable PTSD.
  1.19. *Scale of Ethnocultural Empathy (SEE)*^32^ - a 32-item, self-report measure of how strongly one empathizes with people of other racial and ethnic backgrounds. It provides 4 indices: Empathic Feeling and Expression, Empathic Perspective Taking, Acceptance of Cultural Differences, and Empathic Awareness.
  1.20. *Scales from the Social Psychological Measurements of COVID-19: City, State and Federal Government Response to Coronavirus Questionnaire*^11^ - self-report surveys of beliefs and opinions regarding how city, state, and federal government is, and should be, addressing the COVID-19 pandemic. Includes items related to personal restrictions, punishment for violating restrictions, emotional reactions, advocating for research, economic stimulus, and distrust of public information.
  1.21. *Social Dominance Orientation*^33^ - a 16-item self-report index of preference for social inequality vs. hierarchy attenuation.
  1.22. *Social Network Index (SNI) Extended (current & 2019 version)*^34^ - a self-report questionnaire used to quantify the extent of one’s social connections during a specific timeframe. Outcome variables include: a) Number of High-Contact Roles/Network Diversity (number of social roles in which the respondent has contact with one person or more at least once every 2 weeks; maximum is 12 including spouse, parent, child, child-in-law, close relative, close friend, church/temple member, student, employee, neighbor, volunteer, and group member), b) Number of People in Social Network (measures the total number of people which whom respondent maintains contact at least once every 2 weeks - reflecting overall network size), and c) Number of Embedded Networks (measures the number of different groups these contacts belong to, reflecting network complexity; maximum is 8 including family, friends, church/temple, school, work, neighbors, volunteering, and groups).
  1.23. *State Trait Anxiety Inventory (STAI, state & trait)*^35^ - a self-report questionnaire that differentiates between the temporary condition of “state anxiety” and the more general and long-standing quality of “trait anxiety.” State anxiety is characterized by feelings of apprehension, tension, nervousness, and worry. Elevated trait anxiety is characterized by an overall pattern of increased arousal response to physical danger and psychological stress. Trait anxiety is typically high in people with depression or other psychiatric conditions.
  1.24. *Very Short Authoritarianism Scale (VSA)*^36^ - a six-item self-report questionnaire that assesses right-wing authoritarianism. VSA scores have been shown to predict other factors including nationalism, ethnocentrism, political orientation, political party/candidate support, attitudes towards ingroups or outgroups and anti-minority bias. The measure addresses three facets of authoritarianism: authoritarian submission, authoritarian aggression and conventionalism.
2. Self-report questionnaires - Measures created by COVID-Dynamic Team
  2.1. COVID & Protest Questionnaires
    2.1.1. *COVID Disgust & Fear* - brief questionnaires regarding strength of current disgust (9 items) and fear (7 items) responses when faced with various COVID-related events.
    2.1.2. *COVID Expectation Bias* - instrument that aims to identify bias in predicting 3 COVID-related outcomes (diagnosis of COVID-19, severe illness from COVID-19 and severe financial distress). For each outcome, the participant is asked to rate how likely this is for a randomly selected group. Groups are designated according to race/ethnicity, socioeconomic status, and other factors (elderly, immigrants, healthcare workers, etc …).
    2.1.3. *COVID Experiences* - questionnaire used to identify COVID-related experiences (e.g., diagnosis, illness, treatment), impact of pandemic on daily activities, engagement with news media, and judgment of leadership responses to the pandemic.
    2.1.4. *COVID Explicit Norms* - participants rate the importance of 3 specific COVID-19 prevention tactics (social isolation, social distancing and use of personal protective equipment).
    2.1.5. *COVID Mask Questions* - self-reported description of mask-usage before and during the pandemic, as well as beliefs regarding mask-usage to address the pandemic.
    2.1.6. *COVID Public Policy Knowledge* - a 12-item questionnaire that assesses the participants’ knowledge of current state-enacted COVID-related policies.
    2.1.7. *COVID Theories* - very brief questionnaire about beliefs regarding the existence and origins of COVID-19.
    2.1.8. *COVID Vaccination* - survey created to assess experiences, attitudes, and beliefs related to COVID-19 vaccines.
    2.1.9. *COVID Vignettes – Implicit Norms* - implicit assessment of how participants attribute responsibility/blame regarding the spread of COVID-19. Participants read one out of 9 brief vignettes describing how the behavior of two people may contribute to transmitting COVID-19 to a pharmacist and then answer questions regarding responsibility of the people in the vignette.
    2.1.10. *Post-Election 2020* - survey created to characterize voting behavior in the November 2020 US presidential election, as well as beliefs and attitudes regarding voting and the election outcome.
    2.1.11. *Protest Surveys (Race)* - surveys created to assess experiences, attitudes, and beliefs related to the racial protests that occurred in June 2020. Peaceful protests, looting, and violence are assessed independently.
    2.1.12. *Protest Survey (Election)* - survey created to assess experiences, attitudes, and beliefs related to the 2020 US presidential election-related protests that occurred in January 2021.
    2.1.13. *COVID Memories* – additional one-time survey created to assess spontaneous memory of events that occurred between March and December 2020.
  2.2. Unstandardized/ Experimental Measures
    2.2.1. *Personal background at enrollment* - demographic questionnaire administered at enrollment.
    2.2.2. *Personal background updates* - questions regarding relationship status, work changes, finances, political activities/affiliations, psychological treatment, religion, and current stressors.
    2.2.3. *Consensus Task* - questionnaire that assesses individual values, beliefs, and preferences across a wide range of topics including COVID-19, social issues, political policies, and hobbies, among others.
    2.2.4. *Emotion Space* - self-report questionnaire regarding current emotional experience.
    2.2.5. *Egocentric-Allocentric Task* - perspective tasking task in which participant responds to spatial questions regarding a photo of a political leader.
    2.2.6. *Monthly Substance Use Questionnaire* - survey created to assess substance-use frequency during the prior month.
    2.2.7. *Race Thermometer* - participants use a slider to indicate how they feel toward 6 racial/ethnic groups: Black/African Americans, Asian Americans, Hispanic/Latinx Americans, White/Caucasian Americans, Chinese People, and European People. The scale ranges from 0 (Very Cold or Unfavorable) to 100 (Very Warm or Favorable).
    2.2.8. *Responsibility Vignettes* - implicit assessment of how participants attribute responsibility/blame in a common, low-risk social interaction. Participants read one out of 3 brief vignettes describing how the behavior of two people in a reception area inconveniences an important client that arrives after them, then answer questions regarding responsibility of the people in the vignette.
    2.2.9. *Restriction Severity* - scale that assesses how severe an individual finds various restrictions that may be enforced or encouraged during the pandemic.
    2.2.10. *Self-Continuity*^37^ - this measure asks individuals to rate changes in self-perception, behavior, and emotional experiences resulting from events during 2020. Participants retrospectively rated changes in these 3 domains as of March, June, and September 2020.
    2.2.11. *Verbal Self-Fluency*^38,39^ - in this measure, individuals are asked to provide up to 30 statements that “describe you as a unique individual.” These free text responses are evaluated for total number of self-statements provided.
      In addition to questionnaires, participants completed a series of computer-based tasks designed to assess implicit social attitudes towards race (specifically Black/White/Asian) and pro-sociality, as well as estimations of COVID-related trustworthiness, and social decisions related to pro-sociality, group-cohesion, and altruism. The specific tasks included were:
3. Tasks
  3.1 *Pro-social Implicit Association Task (IAT)* - a version of the standard implicit association test^40^ that assesses implicit associations between self-identity (ME/THEY words) and pro-sociality (SERVICE/PROFIT words)^41^.
  3.2 *Multi-category Brief Implicit Association Task (BIAT)* - a brief form of the implicit association test^42,43^ that assesses implicit associations in 3 subtests (administered in random order): (1) self-identity (ME/THEY words) and pro-sociality (SERVICE/PROFIT words); (2) evaluative (GOOD/BAD words) and White/Black People (WHITE/BLACK faces); and (3) evaluative (GOOD/BAD words) and White/Asian People (WHITE/ASIAN faces).
  3.3 *Multi-race Affective Misattribution Procedure (AMP)* - this adaptation of the Multi-race Affect Misattribution Procedure^44^ assesses evaluative (PLEASANT/UNPLEASANT) implicit associations with different races (Asian, Black, and White).
  3.4 *COVID-related Trustworthiness Rating Task* - in this adaptation of the Trustworthiness Rating Task^45^, participants are shown pictures of Asian/Black/White/other race faces and are asked to rate “How much do you trust this person to act responsibly with respect to the COVID-19 pandemic?” on a scale from 1 (not-at-all) to 9 (completely).
  3.5 *Altruism Task*^46^ - in this adaptation of the Altruism Task, participants make monetary decisions to distribute money equally or unequally (generous/selfish) amongst themselves and partners who vary with regard to age, race, occupation, and political identity.
  3.6 *Public Goods Game*^47,48^ - a standard task from behavioral economics that assesses cooperation and self-interest and group cohesion in an anonymous group fund-sharing scenario. Instructions for the public goods game were adapted from Wills *et al*.^48^.
    A sample survey (including a randomly selected subset of items from the questionnaires and tasks) is available for exploration (https://adolphslab.github.io/CVD-DYN_datarelease/). Survey and task administration files (Qualtrics.qsf and .pdf files, and task html-code) are publicly available for all unpublished surveys (i.e., excluding BDI-II, NEO-FFI, and STAI) and tasks on the Open Science Framework website (questionnaires: https://osf.io/nhm2v/; tasks: https://github.com/adolphslab/CVD-DYN_datarelease/tree/main/exp_tasks).
4. External measures In addition to data collected from participants, we aggregated data from a variety of external sources and linked the external data to the COVID-Dynamic data-collection-schedule, and participants’ individual wave-by-wave physical locations (US-state and county). See https://github.com/adolphslab/CVD-DYN_datarelease/tree/main/external_data for details on data-extraction and processing. The curated external measures can be used to understand the participant data in context, they include:
  4.1 Wave-by-wave public US-state-and-county-level 7-day COVID-19 statisticsWe provide averages over the week preceding each wave’s release date of cumulative and new daily confirmed COVID-19 cases and deaths, day-to-day changes (7-average of day-to-day differences) in new cases and deaths extracted from the daily updated public New-York Times repository^49^ (https://github.com/nytimes/covid-19-data) at the US-state and county level.
  4.2 Wave-by-wave legal restrictions imposed to mitigate the spread of COVID-19
    4.2.1 County-level restrictions: These include stay-at-home-orders, gathering bans, mask mandates, and restaurant and bar closures that were in place during the day each wave was opened to participants. We retrieved these data from public Center for Disease Control (CDC) repositories^50–54^.
    4.2.2 US-state-level restrictions: On the state-level we gathered information on stay-at-home or shelter-in-place orders, limits on gathering size, mask mandates, restrictions on business or retail, and closures of public parks and playgrounds. We assembled this data by evaluating the language in executive orders, public health orders, and official statements by parks departments in place during each wave. Specifically, we measured whether the restrictions were mandatory (often indicated by the imposition of fines or punishments or language stating that citizens “shall” or “must” comply) or suggested (often indicated by language stating that the restrictions were “recommended”). All were coded at the state level. If different counties imposed different restrictions, we noted the predominant restrictions in the state for that wave. Where these statements were uninformative, we supplemented with contemporaneous news headlines and by checking live resources maintained by the *New York Times*^55^ or National Public Radio^56^. Masks and stay-at-home orders were coded as mandatory if an official executive or public health order required them or imposed a fine for violation and recommended if that state’s governor or public health department encouraged compliance, either in an official order or in a press conference or online but did not formally impose the restriction. Size restrictions of gatherings were coded on a scale of 1 to 11, borrowing aspects of MultiState’s methodology^57^. We assigned larger scores to states that recommended rather than mandated the restriction, as assessed by language used in the executive order; instituted larger actual gathering sizes; or opted to delegate gathering restrictions to municipalities rather than setting a statewide limit. Parks were coded in one of three options, all based on official statements from governors, public health departments, or park departments: either fully closed to all visitors, partially closed--for instance, to camping or other activities requiring public facilities–or open. Playgrounds were coded separately as either open or closed, as this was largely left to municipalities to regulate, and official park statements often neglected playgrounds.
  4.3 Monthly US-state and county-level civil unemployment ratesWe retrieved seasonally adjusted monthly state and county-level unemployment numbers from the U.S. Bureau of Labor Statistics’ public data API (https://www.bls.gov/bls/api_features.htm).
  4.4 Wave-by-wave US-state and county-level anti-racism political crowd data from the Crowd Counting Consortium (CCC), which collates publicly reported occurrences of political crowds in the United States (e.g., marches, protests, riots, or demonstrations)^58^. We extracted anti-racism crowd data from the CCC database by selecting events whose descriptions contained ‘raci’. Counter-protest crowd events, i.e., events with ‘against anti-racism’ in their descriptions, were removed.
    Figure 1 provides an overview of COVID cases, key events in the US, stay-at-home restrictions, unemployment rates, anti-racist crowd data, and other pertinent information.

## Data Records

All deidentified data is available on the OSF data sharing platform^59^ (10.17605/OSF.IO/KEX8Y). Questionnaire-based measures are provided in a single long-format csv-file, i.e., rows = participants × waves, columns = questionnaire items. Each task is available as an individual long-format csv-file, i.e., rows = participants × waves × task-trials, columns = trial-by-trial task items. All state-level external data are provided in a long-format csv-file, i.e., rows = US-state × waves, columns = external measures. All survey items, i.e., questionnaire and task-based data, and all data quality variables (see *Technical Validation* for more details) are detailed in a single data dictionary listing possible responses for each item and scores generated for each measure; external measures are described in a second data dictionary (10.17605/OSF.IO/KEX8Y)^59^.

Sensitive geographic information – i.e., data with higher geographic resolution than participant state of residence, such as zip-code and nearest town of residence – bears the risk of identifying individual participants, as does data derived from sensitive location data – i.e., county of residence and county-based external measures – and is excluded from the public data^60^. To access sensitive geographic information researchers must establish a formal relationship with the COVID-Dynamic Team. First, they have to contact the COVID-Dynamic Team by completing a brief form (https://osf.io/7wq9t/). Second, researchers will be asked to provide project approval by their respective Internal Review Board and to establish a data use agreement with the Caltech Internal Review Board.

## Technical Validation

### Demographic representativeness of the core sample

The core sample of 1177 participants (51.23% female, median age = 39.38; age range = 18–77) includes participants from all 50 states. Figure 3 compares the core sample’s demographic distributions to the overall US population^61,62^, including: US state of residence at Wave 1 (top left), distribution of age and birth sex at Wave 1 (top right), political self-identification (bottom left), race (bottom middle), and ethnicity (bottom right) at Wave 1. Figure 3 suggests both overlap and differences between the core sample and US-population demographics on the various variables of interest. The distribution of participants’ state of residence was reflective of the overall US population with a few notable instances of over-representation (California, Oregon, and Washington) counterbalanced by more distributed under-representation.

The participant distribution of sex at birth reflected that of the US population. As expected, likely due to age-related differences in internet usage, older participants (>45) are systematically under-represented in our sample, despite our efforts during recruitment (see *Methods*). With regards to political party affiliation, our sample contains an over-representation of Democrats with corresponding under-representation of Republicans and Independents. Political leanings (liberal/conservative) were skewed according to party affiliation, i.e., Democrats leaned more liberal and Republicans more conservative, with Independents endorsing a more balanced range of leanings. Finally, regarding race and ethnicity, White, Asian, and non-Hispanic/Latinx participants were over-represented in our core sample relative to the US population, whereas Black, Multiracial and Hispanic/Latinx participants were under-represented.

#### Data raking

For a more detailed analysis of the sample’s demographic representatives and to provide resources to “correct” for unrepresentative sample demographics we computed *raking weights* on a sub-set of demographic variables. Data raking is a common post-stratification method used by survey researchers to account for biases in sample demographics to make the sample more representative of the relevant population—the US population in this case. For researchers who wish to correct the sample in this manner, we provide raking weights for sex, age bracket, race and ethnicity for the core sample (N = 1177) according to their marginal distributions as given in the American Community Survey 5-Year Data^61^. Weights were computed in R^63^ using the anesrake package^64^ with a 5% discrepancy limit and no trimming cap. Following the recommendations of Battaglia *et al*.^65^, we collapsed variable categories with less than 5% in the sample (race: American Indian/Alaska Native (0.09%), Native Hawaiian or Other Pacific Islander (0.09%), Multiracial (3.14%), Other (0.42%) and Prefer not to disclose (0.33%); age: 18–19 (3.2%; collapsed with 20–24; Note: US-census age data is provided in 5-year age brackets, i.e., our youngest participants (18–19) fall within the 15–19 bracket but cover only a small subset of that bracket) and all age brackets ≧ 65 (summing to 5.3%). Further, we imputed missing ethnicity data (0.99% Prefer not to disclose) by randomly sampling from the two variable categories (Hispanic or Latinx and Not Hispanic or Latinx).

Despite the clear underrepresentation of conservative and Republican participants in the COVID-Dynamic dataset we refrained from raking with respect to political orientation and ideological leaning. Obtaining an accurate representation of the political landscape has become challenging in recent years^66^. Recent research suggests this may be because the current political landscape in the US is rapidly changing and a more fine-grained categorization beyond party-affiliation might be needed to capture political identity^67^. As we lack a reliable and timely target estimate of the current political/ideological distribution across the US-population, we decided not to rake with respect to political/ideological orientation to avoid inaccurate and potentially misleading weights. Researchers intending to use the data to explore questions that specifically target the influence of demographic and political disparities or that might be confounded by bias in demographic characteristics should do so with the utmost caution and bear in mind the limitations of the data.

#### Sample differences and raking weights

Raking weights revealed considerable age differences between our core sample and the US population. While 45 to 49-year-old individuals are most overrepresented in the sample (+11.8%), residents over 60 years old are the most underrepresented (−17.8%). Across races, sample-Census differences were largest in the Asian (−5.4%) and Black (+5.5%) groups. Ethnicity (Hispanic or Latinx/ not Hispanic or Latinx) was misrepresented by (+/−7.9%). The US-population’s female/male proportion was well represented in the sample (+/−1.7% female/male). Participant-specific raking weights (mean = 1; SD = 0.89; min = 0.3; max = 5) are provided with the data release for those researchers who wish to utilize them. The US-Census target proportion and the core sample’s original proportion across age, sex, race, and ethnicity as well as summary weights for each demographic variable are presented in Tables 1–4.

**Table 1:** Summary Weights for Core Sample Data Raking by Sex assigned at Birth.

sex assigned at birth	target prop. (US census)	unweighted N (core sample)	unweighted prop. (core sample)	wtd N	wtd prop.	change in prop.	resid. disc.	orig. disc.
**female**	0.508	603	0.512	624.414	0.531	0.018	−0.023	−0.004
**male**	0.492	574	0.488	552.586	0.469	−0.018	0.023	0.004
**sum**	1	1177	1	1177	1	0.036	0.045	0.009

NOTE: prop. = proportion; wtd = weighted; resid. disc. = residual discrepancy after weighting; orig. disc. = original discrepancy before weighting. The target proportion (US-census data) is based on the American Community Survey 5-Year Data^61^.

**Table 2:** Summary Weights for Core Sample Data Raking by Age Range.

age range	target prop. (US census)	unweighted N (core sample)	unweighted prop. (core sample)	wtd N	wtd prop.	change in prop.	resid. disc.	orig. disc.
**18–24**	0.093	191	0.162	109.011	0.093	−0.07	0	−0.07
**25–29**	0.095	170	0.144	112.17	0.095	−0.049	0	−0.049
**30–34**	0.09	119	0.101	105.851	0.09	−0.011	0	−0.011
**35–39**	0.086	34	0.029	101.111	0.086	0.057	0	0.057
**40–44**	0.083	128	0.109	97.952	0.083	−0.026	0	−0.026
**45–49**	0.087	241	0.205	102.691	0.087	−0.118	0	−0.118
**50–54**	0.09	82	0.07	105.851	0.09	0.02	0	0.02
**55–59**	0.09	85	0.072	105.851	0.09	0.018	0	0.018
**60–100**	0.286	127	0.108	336.511	0.286	0.178	0	0.178
**sum**	1	1177	1	1177	1	0.546	0	0.546

NOTE: prop. = proportion; wtd = weighted; resid. disc. = residual discrepancy after weighting; orig. disc. = original discrepancy before weighting. The target proportion (US-census data) is based on the American Community Survey 5-Year Data^61^. To account for the difference in the included age range in the ACS’ and our sample’s lowest age bracket (ACS: 15–19; survey sample: 18–19) we used the census 20–24 bracket as target for the collapsed 18–24 age bracket in the sample.

**Table 3:** Summary Weights for Core Sample Data Raking by Race.

race	target prop. (US census)	unweighted N (core sample)	unweighted prop. (core sample)	wtd N	wtd Prop.	change in prop.	resid. disc.	orig. disc.
**American Indian/Alaska Native, Native Hawaiian or Other Pacific Islander, Multiracial, other, prefer not to disclose**	0.091	85	0.072	107.214	0.091	0.019	0	0.019
**Asian**	0.054	121	0.103	63.622	0.054	−0.049	0	−0.049
**Black**	0.127	85	0.072	149.629	0.127	0.055	0	0.055
**White**	0.728	886	0.753	856.536	0.728	−0.025	0	−0.025
**sum**	1	1177	1	1177	1	0.148	0	0.148

NOTE: prop. = proportion; wtd = weighted; resid. disc = residual discrepancy after weighting; orig. disc. = original discrepancy before weighting. The target proportion (US-census data) is based on the American Community Survey 5-Year Data^61^.

**Table 4:** Summary Weights for Core Sample Data Raking by Ethnicity.

ethnicity	target prop. (US census)	unweighted N (core sample)	unweighted prop. (core sample)	wtd N	wtd prop.	change in prop.	resid. disc.	orig. disc.
**Hispanic or Latinx**	0.178	119	0.101	209.506	0.178	0.077	0	0.077
**Not Hispanic or Latinx**	0.822	1058	0.899	967.494	0.822	−0.077	0	−0.077
**sum**	1	1177	1	1177	1	0.154	0	0.154

NOTE: prop. = proportion; wtd = weighted; resid. disc. = residual discrepancy after weighting; orig. disc. = original discrepancy before weighting. The target proportion (US-census data) is based on the American Community Survey 5-Year Data^61^.

### Data quality

#### Quality measures

To characterize the data quality, 16 quality indices (described below) were calculated for each wave and each participant and then compared to index-specific thresholds. A summary score for each participant of the percentage of quality indices deemed ‘passed’ was then calculated. Results for each individual index (both index value and the binary pass/fail outcome; 1 = fail) are provided in the data release. Note, these quality metrics were not used for participant exclusion. Participant exclusion was based solely on the criteria detailed in *Participants* and applied only to the first 5 waves. All quality metrics described below are provided with the data and researchers can apply those metrics for exclusion at their own discretion.

Several data quality indices were based on the mean number of consecutive identical responses in questionnaires and tasks (i.e., the mean response-string length). Each participant’s mean response-string length was computed for each questionnaire/task in each wave. Participants with mean response-string length outside the range defined by the 1st quartile-3*IQR and the 3rd quartile + 3*IQR were deemed outliers. The same criterion was applied to identify outliers in response consistency and wave completion time (see data quality index 9: “Response consistency” below).

Data quality indices for each participant and each wave included:

1. Completed less than 50% of waves (i.e., <8 waves).
2. Failed 2 or more attention questions.
3. Wave completion time: From wave 1 to 5, based on group-examination of the distribution of overall wave-specific completion times, the COVID-Dynamic Team determined an adjustable cutoff (in 5-minute intervals) of participant survey completion times that were deemed too brief for completing the survey in good faith. From Wave 5 onwards the lowest cutoff (<25 min) was used to flag low wave completions times.
4. Repetitive response behavior:
  - Mean response-string length deemed an outlier in greater than 50% of all questionnaires with more than 4 items
  - Mean response-string length deemed an outlier in greater than 50% of “core” questionnaires: Positive and Negative Affect Scales (PANAS), State Trait Anxiety Inventory (STAI, state and trait), NEO Five-Factor Personality Inventory (NEO-FFI), Very Short Authoritarianism (VSA), and Scale of Ethnocultural Empathy (SEE)
  - Mean response-string length deemed an outlier in the Multi-race Affective Misattribution Procedure
  - Mean response-string length was deemed an outlier in the Altruism Task (from Wave 4 on, prior to Wave 4 response key associations were not randomized and long response strings were considered plausible behavior).
5. Response speed:
  - More than 10% of RTs < 300 ms in COVID-related Trust Rating Task
  - More than 10% of RTs <300 ms in Altruism Task
  - More than 10% of RTs <300 ms in Multi-race Affective Misattribution Procedure
6. Task data quality:
  - Excluded from Pro-social Implicit Association Task based on recommended IAT analysis criteria^68^
  - Excluded from Multi-category Brief Implicit Association Task based on recommended BIAT analysis criteria^43^
7. Free text responses: Valid free text responses were defined as responses that included at least one verb or noun per response. The number of nouns and verbs was automatically extracted using the Natural Language Toolkit^69^.
  - Valid responses present in less than 50% of the questions asking participants to list stressors in their life.
  - Valid responses present in less than 50% of the questions asking participants to list memorable news items.
8. Frequency of missing (i.e., NA) responses greater than 50% in questions that include NA response options (e.g., “does not apply”, “prefer not to disclose”, or similar). Table 6 summarizes NA-responses across waves.
9. Response consistency: Response consistency was quantified via the mean difference score between regular and reverse scored items in STAI, SEE, and the Disgust Scale-Revised (DS-R) and mean score differences between PANAS positive and negative items and PANAS positive and PSS scores. Participants that were deemed an outlier (defined via the IQR, see above) in less than 50% of included comparisons are considered to pass this quality measure.

#### Data quality summary

To summarize overall data quality, we calculated the number of participants that passed increasing percentages of our 16 data quality indices (Note: not all quality indices are applicable in each wave) per wave (Table 5). In all waves, most participants (max = 74.86%; min = 57.74%) passed all data quality metrics. Pass rates increased as the percentage threshold decreased, plateauing between around 70% thresholds. Participant- and wave- wise scores for all quality indices as well as flags for missing task data are provided in the data release.

**Table 5:** Wave-by-Wave Performance on Data Quality Metrics.

wave	N quality metrics	pass 100%	pass >90%	pass >80%	pass >70%	pass >60%	pass >50%	pass >40%	pass >30%	N subj.
**1**	12	43.29% (778)	80.24% (1442)	94.77% (1703)	98.39% (1768)	99.61% (1790)	99.94% (1796)	100% (1797)	100% (1797)	1797
**2**	14	45.19% (677)	78.37% (1174)	92.12% (1380)	98.53% (1476)	99.73% (1494)	99.87% (1496)	100% (1498)	100% (1498)	1498
**3**	13	49.73% (727)	82.08% (1200)	93.98% (1374)	98.22% (1436)	99.66% (1457)	99.93% (1461)	100% (1462)	100% (1462)	1462
**4**	15	56.65% (788)	87.35% (1215)	96.76% (1346)	99.64% (1386)	99.93% (1390)	100% (1391)	100% (1391)	100% (1391)	1391
**5**	13	58.11% (745)	85.34% (1094)	95.32% (1222)	98.44% (1262)	99.84% (1280)	100% (1282)	100% (1282)	100% (1282)	1282
**6**	13	66.98% (789)	89.81% (1058)	96.69% (1139)	98.73% (1163)	100% (1178)	100% (1178)	100% (1178)	100% (1178)	1178
**7**	15	56.57% (659)	81.12% (945)	92.1% (1073)	99.06% (1154)	99.57% (1160)	100% (1165)	100% (1165)	100% (1165)	1165
**8**	13	66.27% (729)	91.09% (1002)	97.45% (1072)	99.27% (1092)	100% (1100)	100% (1100)	100% (1100)	100% (1100)	1100
**9**	13	72.47% (758)	93.31% (976)	97.32% (1018)	99.33% (1039)	100% (1046)	100% (1046)	100% (1046)	100% (1046)	1046
**10**	13	66.57% (685)	91.35% (940)	97.47% (1003)	99.13% (1020)	99.9% (1028)	100% (1029)	100% (1029)	100% (1029)	1029
**11**	13	66.57% (657)	91.08% (899)	97.67% (964)	99.29% (980)	99.9% (986)	100% (987)	100% (987)	100% (987)	987
**12**	13	69.21% (645)	92.81% (865)	97.75% (911)	99.36% (926)	100% (932)	100% (932)	100% (932)	100% (932)	932
**13**	13	67.73% (638)	92.25% (869)	97.56% (919)	99.15% (934)	100% (942)	100% (942)	100% (942)	100% (942)	942
**14**	13	65.75% (597)	91.74% (833)	97.03% (881)	99.12% (900)	99.78% (906)	99.89% (907)	99.89% (907)	100% (908)	908
**15**	13	66.22% (596)	91.33% (822)	97.89% (881)	99.44% (895)	100% (900)	100% (900)	100% (900)	100% (900)	900
**16**	13	66.89% (586)	91.89% (805)	97.83% (857)	99.54% (872)	100% (876)	100% (876)	100% (876)	100% (876)	876

For each wave (rows) the count of participants that completed that wave is displayed in the last column. The first column shows the subset of the 16 data quality metrics that are applicable in each wave. The remaining columns show the percentage (and count) of participants per wave that passed decreasing percentages (100% to 30% in decrements of 10%) of our 16 data quality indices.

**Table 6:** Count of NA-Responses.

wave	N NA-questions	>1 NA resp	>4 NA resp	>7 NA resp	mean NA resp	max NA resp	N participants
**1**	8	134	14	0	0.28	7	1797
**2**	8	108	12	0	0.29	7	1498
**3**	18	117	18	1	0.34	17	1462
**4**	18	114	15	1	0.33	8	1391
**5**	30	388	132	41	1.41	20	1282
**6**	30	398	166	61	1.69	19	1178
**7**	18	27	5	0	0.12	7	1165
**8**	18	60	8	3	0.25	15	1100
**9**	30	335	166	71	1.82	21	1046
**10**	18	59	10	2	0.26	8	1029
**11**	18	53	11	1	0.25	8	987
**12**	18	48	13	2	0.25	10	932
**13**	18	46	15	1	0.24	10	942
**14**	18	42	14	3	0.25	18	908
**15**	18	40	7	1	0.23	8	900
**16**	18	48	10	4	0.28	11	876

For each wave (rows) the count of participants that completed that wave is displayed in the last column. The first column contains the number of items for each wave that did not force a response or included an NA-response options. The three subsequent columns show the count of participants that provided more than 1, 4 or 7 NA response. The next two columns summarize the mean and maximum number of NA responses across participants.

### Survey-data overview

**Fig. 4:**
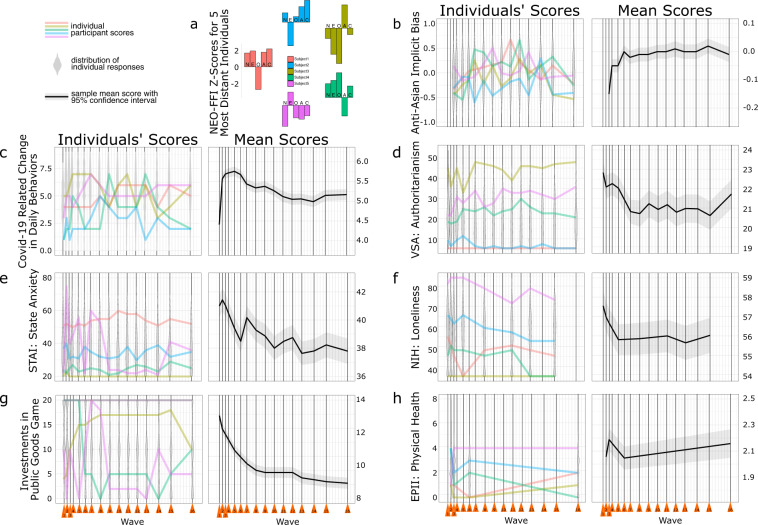
Group (core sample N = 1177) and Individual (N = 5) Temporal Trends from an Example Variable in Each Domain Assessed. (**a**) NEO-FFI scores (five-factor personality inventory - N: Neuroticism, E: Extraversion, O: Openness to experience, A: Agreeableness, C: Conscientiousness) from five participants with maximally distant personality profiles. (**b**–**h**) Results at the individual- (5 exemplary participants; left) and group- (core sample; right) levels for a single measure from each of the study domains. Measure scores are represented on the y-axis, time since beginning of data collection (in weeks) is represented on the x-axis). Orange triangles indicate weeks in which data were collected. In “Individuals’ Scores” graphs, grey-shaded violin plots show the distribution of all 1000 + participant’s responses in each wave, overlaid colored lines indicate the individual scores from each of the five participants described in panel A. In “Average Scores” graphs, the black line indicates the mean response across all 1000 + participants in each wave, with 95% confidence intervals denoted by the shaded region. Panels B-H present one example measure from each domain. In each panel description below, the meaning of higher values for the presented score is given in parentheses: (**b**) implicit attitudes, Asian/White + Good/Bad brief implicit association test (IAT), IAT D score (higher anti-Asian attitudes); (**c**) daily life impact, COVID-19 related changes in daily behaviors, sum total of 9 items (greater changes in behavior); (**d**) explicit attitudes and beliefs, very short authoritarianism (VSA) scale, VSA raw summary score (higher authoritarian tendencies); (**e**) emotion and mental health, State-Trait Anxiety Inventory state scale (STAI-state), STAI-state raw summary score (higher state anxiety); (**f**) social behaviors - National institutes of Health Loneliness scale, T-score (increased loneliness); (**g**) decision making, Public Goods Game group-investments, tokens (amount invested with the group); (**h**) physical health - Epidemic-Pandemic Inventory (EPII) physical health total, sum of 8 items (higher number of non-COVID health problems endorsed).

To illustrate the richness of the data, and in particular the breadth of included measures and the variation over time both across and within individuals, we selected a subset of representative measures for visualization. Figure 4 depicts results for the core sample of 1177 participants and for five participants with maximally divergent personality profiles (see below for methodological details) for one representative measure (i.e., item or summary score) from each measurement domain assessed in the study (i.e. daily life impact, attitudes and beliefs, emotion and mental health, physical health, social behaviors, decision making tasks, and implicit attitudes; note: demographics are omitted as these are visualized in detail in Fig. 3). For each measure (Fig. 4b–h) and wave (x-axis), the distribution of individual scores (left panels) is depicted in a violin plot with the individual scores from the five exemplary participants superimposed (NEO-FFI profiles for each of the five individuals are shown in Fig. 4a). The group-level plot for each measure (right panels) shows the mean response across all participants in each wave. Note: not all measures were collected every wave. Collectively, the data in Fig. 4 demonstrate there exist substantial and meaningful variations in the data across the waves, both on average across participants and for individual participants over time. Hence, each measure has a clear pattern of evolution on average across participants over the displayed time. Moreover, individual participants with distinct personalities and life situations had distinct trajectories within each measure.

#### Identification of distinct individual participants for visualization

A key strength of this longitudinal dataset is that it facilitates studying individual differences in temporal variation of responses. To illustrate this potential, data from 5 individual participants from the group of participants that completed all 16 waves were selected for visualization in Fig. 4. The particular set of 5 participants was selected using a maximum variation sampling procedure^70^, which sampled participants by maximizing the sum of Euclidean distances between their scores on the five NEO-FFI subscales reflecting their personality traits. The first participant in a set was drawn randomly. Subsequent participants were selected so that each new participant maximized the Euclidean distances from the previously selected participants in the 5-dimensional personality vector space. We repeated the sampling process for all possible initializations and selected the specific sample with the maximum sum of Euclidean distances. The patterns of each of the selected 5 participants’ NEO-FFI scores are illustrated in Fig. 4a.

#### Shiny app

In addition to selected example variables, we provide a freely available online visualization tool (http://coviddynamicdash.caltech.edu/shiny/coviddash/) aimed to provide researchers with a wider glimpse at the dataset. This tool was built using the Shiny-app framework^71^ in R. Users have the option to filter the sample using demographic variables, visualize group mean temporal trends on a subset of measures included in this study, and examine within-wave Pearson correlations of any two measures in a selected wave. We encourage all users of the data to explicitly detail any data-exploration using this tool preceding formal analyses and hypothesis testing in their pre-registrations and publications.

### Sample loss

**Fig. 5:**
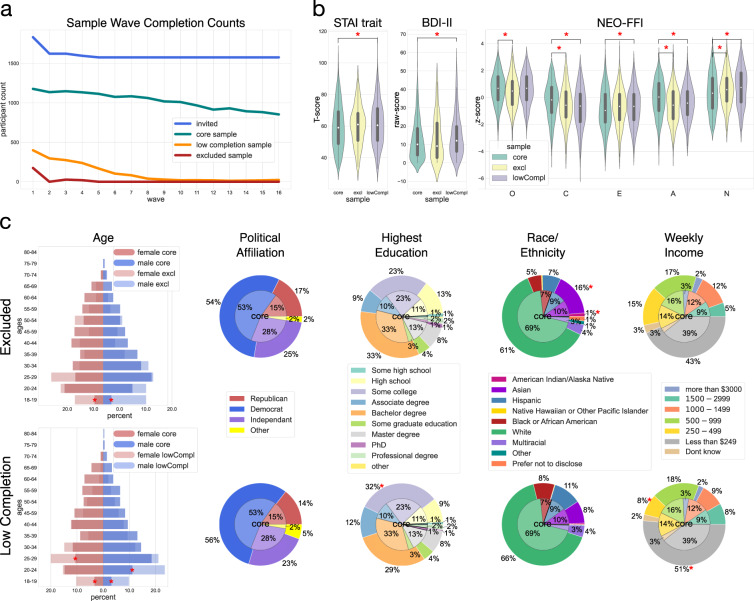
Psychological and Demographic Comparison of Excluded (N = 221) and Low-Completion Rate (N = 399) Groups to the Core Sample (N = 1177). (**a**) Count of participants invited via Prolific to complete each wave (W1: N = 1831; W16: N = 1576); and counts of participants included in the core sample (N = 1177), the low-completion rate (N = 399), and the excluded (N = 221) group that completed each wave. To test for demographic and psychological sample selection biases we compared the excluded and low-completion rate groups to the core sample on several psychological and demographic measures. Significant differences between the core sample and the excluded or low-completion group, respectively, are marked with red asterisks (see Table 7 through Table 9 for details). (**b**) We inspected seven psychological variables: trait anxiety (STAI trait; panel B left), depression (BDI-II; panel b center), and personality (NEO-FFI subscales N = neuroticism, E = extraversion, O = openness, A = agreeableness, C = conscientiousness; panel b right). Violin plots show score-distributions grouped by measures and participant groups; white central dots mark the median-values, grey central markers indicate the interquartile range. (**c**) The excluded and low-completion group (panel C-bottom row) were compared to the core sample on five demographic variables: age, political affiliation, education, race/ethnicity (note: race and ethnicity were collapsed), and income. Butterfly charts superimpose age and sex in the core sample with the excluded and low-completion rate samples. Nested pie charts show all other demographic variables with the core sample’s proportions presented in the center, and the comparison group (excluded: panel C top row; low-completion rate: panel C bottom row) in the outer circles. For readability, labels <1% were dropped from the pie charts.

All within-sample longitudinal assessments suffer from sample loss, i.e., survey-participants dropping out or excluded from longitudinal participation (Fig. 5a). To understand the factors that contributed to sample loss, we characterized participants that did not continue with the longitudinal study by comparing three participant sub-samples: (1) excluded participants - those who were excluded from further participation (Waves 1–5 only) based on the exclusion criteria listed in the participants section; (2) participants with a low-completion rate - individuals that were not excluded, but completed less than 8 out of 16 waves; and (3) the core sample of participants - those who do not fall within the excluded or low-completion rate categories.

**Fig. 6:**
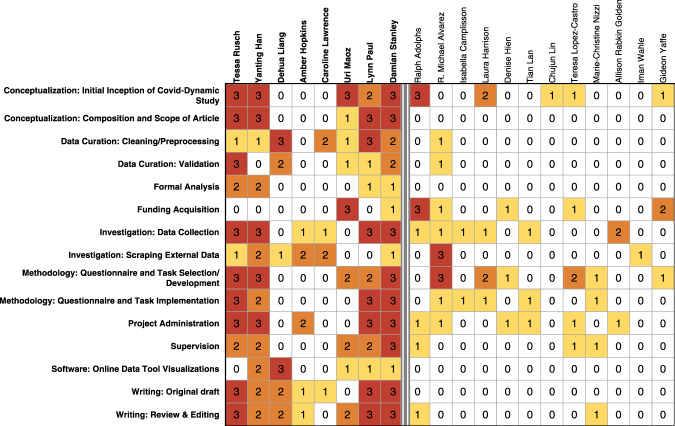
Contribution Table. The main authors’ contributions are displayed in the left section of the table in the same order as the author list. The contributions of COVID-Dynamic Team members that are not listed individually in the author list are displayed in the right section of the table in alphabetical order. Contribution strengths are indicated numerically: 0 (no contribution), 1 (support), 2 (medium), 3 (lead); and color-coded with darker colors indicating stronger contributions.

#### Demographic and psychological assessment

To assess if the participants in the excluded (*N* = 221) and low-completion rate (*N* = 399) groups differed in meaningful ways from the core sample (*N* = 1117), we compared the two omitted groups to the core sample on several demographic and psychological variables. Demographic measures include age, birth sex, political party self-identification, highest education, weekly income bracket, and race and ethnicity. Psychological variables comprise measures of anxiety (STAI-trait), depressive symptoms (Beck Depression Inventory-II; BDI-II), and personality (NEO-FFI scores).

For each of the omitted groups (excluded and low-completion rate), we quantified the difference between that group and the core sample by calculating the Euclidean distance between the samples using vectors representing the categories of each demographic factor, and the difference in mean scores for each psychological factor. The true Euclidean distances (demographic measures) and mean difference scores (psychological measures) between the omitted groups and the core sample were compared against bootstrapped distributions of distances and mean scores of sub-samples drawn from the core sample (N bootstrapped samples = 10,000; N participants in bootstrapped samples [sampled without replacement] = N participants in excluded group/ low-completion rate group). True Euclidian distances and mean score differences between the excluded/low-completion rate group and the core sample outside the 2.5^th^ and 97.5^th^ percentiles of the respective bootstrapped distributions show significant differences between core and omitted samples. Significant differences between core- and omitted- demographics were further examined using post-hoc two-proportion z-tests.

#### *Demographic characteristics*

Both male and female participants in the excluded and the low-completion rate group differed significantly from the core sample in age, with the core sample including significantly less participants in the lowest age brackets (18–29) than the other two groups. The excluded group further differed from the core sample in its racial and ethnical composition with the excluded group containing more Asian and American Indian/Alaskan Native individuals than the core sample. Participants that completed less than 8 out of 16 waves further differed from the core sample in highest education level and weekly income: The proportion of participants with *some college education* was significantly higher than in the core sample; and more participants from the low-completion group fell in the lowest income bracket (*$250*–*499 per week*) while in the core sample a larger proportion of participants fell in the second lowest income bracket (*$500*–*999 per week*) (see Fig. 5c, Tables 7, 8).

**Table 7:** Distance between Core and Excluded/Low-Completion Rate Participants on Five Demographic Measures.

demographic measure	true dist. core-excl	bootstr. dist. 2.5th perc. core-excl	bootstr. dist. 97.5th perc. core-excl	true dist. core-lowCompl	bootstr. dist. 2.5th perc. core-lowCompl	bootstr. dist. 97.5th perc. core-lowCompl
**age female**	15.267*	2.824	7.339	15.088*	1.598	4.002
**age male**	17.218*	2.749	7.322	16.199*	1.452	3.794
**party**	4.065	1.057	9.236	5.925	0.707	6.110
**education**	6.358	2.363	8.961	11.523*	1.564	5.923
**race/ethnicity**	10.803*	1.442	7.839	4.149	0.978	5.177
**income**	5.925	2.164	8.967	14.513*	1.451	5.943

Bold distance values are significantly different (*p < 0.05; two sided) from the bootstrapped null distributions. NOTE: excl. = excluded; bootstr. = bootstrap; perc. = percentile; compl. = completion. See Fig. 5 for follow-up-comparisons across categories within age, education, race and ethnicity, and income.

**Table 8:** Between participant-sample comparisons within demographic measures.

a							
age female	prop. core	prop. excl.	prop. low compl.	z-value (core/excl.)	p-value (core/excl.)	z-value (core/low compl.)	p-value (core/low compl.)
age male	prop. core	prop. excl.	prop. low compl.	z-value (core/excl.)	p-value (core/excl.)	z-value (core/low compl.)	p-value (core/low compl.)
**18–19**	0.033	0.108	0.103	−3.507	>0.001*	−**3.861**	>0.001*
**20–24**	0.148	0.162	0.154	−0.395	0.693	−0.213	0.831
**25–29**	0.106	0.198	0.200	−2.739	0.006	−**3.398**	0.001*
**30–34**	0.116	0.09	0.149	0.798	0.425	−1.202	0.23
**35–39**	0.088	0.072	0.056	0.548	0.584	1.407	0.159
**40–44**	0.121	0.09	0.123	0.936	0.349	−0.075	0.94
**45–49**	0.109	0.054	0.077	1.781	0.075	1.308	0.191
**50–54**	0.075	0.108	0.062	−1.196	0.232	0.617	0.537
**55–59**	0.08	0.054	0.041	0.936	0.35	1.833	0.067
**60–64**	0.071	0.027	0.015	1.746	0.081	2.913	0.004
**65–69**	0.045	0.027	0.01	0.857	0.392	2.239	0.025
**70–74**	0.008	0.009	0.01	−0.076	0.939	−0.256	0.798
**75–79**	NA	NA	NA	NA	NA	NA	NA
**80–84**	NA	NA	NA	NA	NA	NA	NA
**18–19**	0.031	0.164	0.098	−5.692	>0.001*	−**3.795**	>0.001*
**20–24**	0.111	0.164	0.235	−1.542	0.123	−**4.326**	>0.001*
**25–29**	0.185	0.191	0.211	−0.154	0.877	−0.814	0.416
**30–34**	0.145	0.173	0.123	−0.759	0.448	0.782	0.434
**35–39**	0.131	0.082	0.093	1.43	0.153	1.412	0.158
**40–44**	0.094	0.045	0.078	1.664	0.096	0.671	0.502
**45–49**	0.084	0.055	0.059	1.036	0.3	1.14	0.254
**50–54**	0.064	0.073	0.044	−0.32	0.749	1.058	0.29
**55–59**	0.064	0.027	0.029	1.523	0.128	1.882	0.06
**60–64**	0.038	0.018	0.02	1.052	0.293	1.278	0.201
**65–69**	0.033	NA	0.005	NA	NA	NA	NA
**70–74**	0.014	0.009	NA	0.409	0.683	1.695	0.09
**75–79**	0.005	NA	NA	NA	NA	NA	NA
**80–84**	NA	NA	0.005	NA	NA	NA	NA
*p Bonferroni = 0.003							

b							
highest education level	prop. core	prop. excl.	prop. low compl.	z-value (core/excl.)	p-value (core/excl.)	z-value (core/low compl.)	p-value (core/low compl.)
**PhD**	0.015	0.018	0.005	−0.308	0.758	1.585	0.113
**master’s degree**	0.138	0.086	0.088	2.099	0.036	2.606	0.009
**some graduate education**	0.033	0.05	0.045	−1.222	0.222	−1.107	0.268
**bachelor’s degree**	0.332	0.339	0.291	−0.207	0.836	1.533	0.125
**professional degree**	0.02	0.023	0.018	−0.214	0.831	0.354	0.723
**associate degree**	0.109	0.09	0.123	0.809	0.418	−0.768	0.442
**some college**	0.232	0.24	0.323	−0.254	0.8	−**3.618**	>0.001*
**high school**	0.111	0.136	0.095	−1.045	0.296	0.896	0.37
**some high school**	0.008	0.014	0.01	−0.722	0.47	−0.281	0.779
**other**	0.001	0.005	0.003	−1.326	0.185	−0.803	0.422
*p Bonferroni = 0.005							

c							
weekly income	prop. core	prop. excl.	prop. low compl.	z-value (core/excl.)	p-value (core/excl.)	z-value (core/low compl.)	p-value (core/low compl.)
**more than $3000**	0.034	0.027	0.02	0.523	0.601	1.4	0.162
**$1500 - $2999**	0.095	0.054	0.083	1.96	0.05	0.744	0.457
**$1000 -$1499**	0.128	0.122	0.09	0.25	0.802	2.032	0.042
**$500 - $999**	0.169	0.176	0.185	−0.268	0.788	−0.748	0.455
**$250 - $499**	0.15	0.154	0.083	−0.165	0.869	**3.401**	>0.001*
**less than $249**	0.393	0.434	0.514	−1.142	0.253	−**4.206**	>0.001*
**don’t know**	0.031	0.032	0.025	−0.086	0.932	0.566	0.571
*p Bonferroni = 0.007							

d							
race/ethnicity	prop. core	prop. excl.	prop. low compl.	z-value (core/excl.)	p-value (core/excl.)	z-value (core/low compl.)	p-value (core/low compl.)
**American Indian/ Alaska Native**	0.001	0.014	0.005	−**3.25**	>0.001*	−1.649	0.099
**Asian**	0.101	0.167	0.088	−**2.873**	0.004*	0.778	0.436
**Black or African American**	0.071	0.054	0.083	0.921	0.357	−0.747	0.455
**Hispanic/ Latinx**	0.094	0.072	0.11	1.04	0.298	−0.926	0.355
**Multiracial**	0.031	0.041	0.045	−0.71	0.478	−1.286	0.198
**Native Hawaiian or Other Pacific Islander**	0.001	0.005	0.003	−1.326	0.185	−0.803	0.422
**White**	0.692	0.615	0.662	2.254	0.024	1.144	0.253
**other**	0.004	0.014	0.003	−1.687	0.092	0.488	0.625
**prefer not to disclose**	0.003	0.018	0.003	−2.658	0.008	0.274	0.784
*p Bonferroni = 0.0006							

For all demographic features ((a) age, (b) highest level of education, (c) weekly income, (d) race/ethnicity) that differed significantly between the core and low-completion rate or the core and excluded sample, respectively (see Table 7), we compared the proportion of participants in each feature bracket between the groups using post-hoc two-proportion z-tests (critical p-value Bonferroni corrected for each feature). Each table lists the within group (core, excluded, low-completion rate) distribution of participants across the respective feature brackets (proportion of sample per bracket), and the z- and p-scores for two-proportion z-tests comparing the core sample against the excluded and low-completion rate groups. Significant differences between samples are highlighted in bold. NOTE: prop. = proportion; excl. = excluded; compl. = completion; abs. diff. = absolute difference.

#### *Psychological Characteristics*

With respect to psychological characteristics, the low-completion group scored higher values of trait anxiety (STAI-trait) and depressive symptoms (BDI-II) than the core sample, as well as lower levels of conscientiousness and agreeableness, and higher levels of extraversion and neuroticism in the NEO-FFI. The excluded group endorsed less openness to experience, conscientiousness and agreeableness, and higher levels of neuroticism on the NEO-FFI than the core sample (see Fig. 5b, Table 9).

**Table 9:** Mean Test Scores on Seven Psychological Measures for the Excluded and Low-Completion Rate Groups.

psychological measures	mean core	mean excl.	bootstr. mean 2.5th perc. excl.	bootstr. mean 97.5th perc. excl.	mean low compl.	bootstr. mean 2.5th perc. low compl.	bootstr. mean 97.5th perc. low compl.
STAI trait T-score	59.391	60.353	57.731	61.11	61.198*	58.258	60.532
BDI-II raw	12.721	12.836	11.393	14.061	14.865*	11.838	13.614
NEO-O z-score	0.663	0.388*	0.526	0.801	0.638	0.572	0.757
NEO-C z-score	−0.239	−0.586*	−0.398	−0.078	−0.737*	−0.347	−0.13
NEO-E z-score	−0.87	−0.739	−1.044	−0.695	−0.696*	−0.989	−0.75
NEO-A z-score	−0.075	−0.585*	−0.235	0.084	−0.38*	−0.18	0.03
NEO-N z-score	0.314	0.576*	0.141	0.486	0.725*	0.2	0.428

Bold mean values are significantly different (*p < 0.05; two sided) from the bootstrapped null distribution. NOTE: excl. = excluded; bootstr. = bootstrap; perc. = percentile; compl. = completion; BDI-II = Beck Depression Inventory – II; STAI = State Trait Anxiety Inventory; NEO = NEO-Five Factor Inventory.

## Usage Notes

While the strengths of these data are numerous, we also note certain limitations.

### Absence of ‘*a priori*’ hypotheses

From its inception, the COVID-Dynamic project, and the resulting dataset, was conceived of as archival in nature. The breadth of the project, combined with the limitations of time (at the time of the project’s inception the pandemic had already reached the U.S.) as well as the inability to predict the duration of the pandemic, its direct and indirect impacts, and the societal response (governmental and the general public), rendered the generation of strong ‘*a priori*’ hypotheses an arbitrary endeavor. Instead, within the COVID-Dynamic Team we implemented a structured project registration process (see *Background* *&* *Summary*). In addition, while freely available, users of the dataset are strongly encouraged to add a public project description (with hypotheses, variables of interest, and planned analyses) to the COVID-Dynamic project repository. This will help to ensure that future research is not duplicated and that hypotheses are ‘registered’.

### Absence of pre-pandemic data

The dataset does not contain within-participant pre-pandemic baseline measures apart from retrospective self-reports. Thus, some initial effects (e.g., acute initial increases in anxiety) are likely not captured by these data. However, the period of coverage includes multiple devastating local and national peaks of COVID-19 cases, deaths, and restrictions, as well as many national events (e.g., BLM protests, US-presidential elections), and extensive documentation of personal experiences, yielding ample subjective variance to probe.

### Missing data

There are many reasons for, and various types of, missing data, each of which present unique analysis challenges. The COVID-Dynamic dataset contains five different types of missing data that result from the study’s irregularly-spaced and sparse sampling schedule, the exclusion of inattentive participants, and participant attrition. These are:

1. Timepoints not covered by the COVID-Dynamic study: Due to funding constraints and the unknown duration of data collection, data were collected at irregular expanding intervals. Thus, weeks that were “skipped” (e.g., 2020 calendar weeks 46–49 between Waves 14 and 15) are “missing” from the COVID-Dynamic dataset.
2. Sparsely sampled measures: Due to time (we did not want the survey to become onerous for our participants) and funding constraints, some measures were sampled intermittently (e.g., BDI was presented every other wave). As a result, data from some measures are “missing” for some time points.
3. Excluded participants: To ensure funding was not wasted, in Waves 1 through 5 we excluded participants with poor quality data from further participation. Data from these participants are therefore “missing” from all subsequent waves.
4. Participant attrition: As is common in longitudinal studies, some participation was intermittent and some participants dropped out altogether. As a result, data from these participants was missing for one or more entire waves.
5. Non-responses: Some participants missed or refused to respond to individual questions/measures within a wave, i.e., individual variables are missing for some participants in otherwise complete waves.

There are multiple methods for addressing missing data (e.g., data imputation^72,73^ and advanced linear modeling methods^74,75^), however, their implementation and reliability heavily depend on the target data and the research question at hand. As a result, each type of missing data in the COVID-Dynamic dataset has different implications when considering imputation. We outline some of these below:

1. Timepoints not covered by the COVID-Dynamic study: Estimating data at time points that were not covered by the COVID-Dynamic schedule would require external data that covers the measures and period in question in a comparable pool of participants. To our knowledge, the COVID-Dynamic dataset is unique, and we are unaware of any comparable data suitable for imputing skipped timepoints in an untargeted manner. Certainly, there are datasets that are suitable for imputing different subsets of the variables we collected. However, such imputation approaches should be tailored to specific research questions rather than applied to the dataset as a whole. Moreover, the complex and highly volatile nature of the period over which the COVID-Dynamic data were collected may render the assumptions of imputation invalid.
2. Sparse measures: To some degree, sparse measures can be extrapolated from a combination of other correlated measures collected at the missing time point. However, once again, the choice of source measures strongly depends on the target variable in question and research-question-specific tests are needed to verify the validity of the respective extrapolations.
3. Excluded participants: Participant exclusion criteria were tailored to identify data of very poor quality with a very low signal-to-noise ratio (e.g., from inattentive and fraudulent responders). Therefore, these data should not be imputed.
4. Participants attrition: Data from participants’ missing waves can be imputed from their own preceding and succeeding data as well as other comparable participants. However, as in (2) the specific research question determines the source data (e.g., for specific questions only certain demographics might be relevant). Moreover, even targeted imputation of data for participants that missed certain time points is risky. Participants’ experiences and responses throughout the pandemic were highly variable and idiosyncratic. For example, participants may not miss waves at random (e.g., waves could coincide with negative life events) and thus even their own preceding data or data from similar participants would not be suited for extrapolation. Indeed, we found that overall sample attrition was demographically- and psychologically- biased (see *Technical Validation – Sample Loss*), underscoring the need for caution when considering imputation.
5. Non-responses: Most measures included in the COVID-Dynamic study were “forced response” – i.e., participants could not proceed with the study if they did not provide a response. In total, less than 1% of all variables allowed participants to proceed without responding, and very few participants chose not to respond. Thus, in the COVID-Dynamic dataset, missing data of this type is negligible.

In summary, distinct approaches and assumptions need to be considered for imputation of different types of missing data. Moreover, analysis methods and best practices for imputation are rapidly evolving. For these reasons, we have not performed any general imputation on these data and strongly recommend that any data imputation should be performed cautiously, using the best practices available, adapted to the specific research questions posed by researchers analyzing the data, as well as tested and validated within the respective study. Most importantly, researchers need to be aware of the potential confounds that could arise when performing imputation and how these confounds limit the interpretability of any findings.

### Data quality and sample biases

Additional limitations, particularly prevalent in anonymous online and longitudinal studies, include poor data-quality and spurious results driven by inattentive or fraudulent responders^76,77^, as well as recruitment and retention biases. To ensure high data-quality, participants who were obviously inattentive or responded fraudulently in early waves were excluded. We also conducted detailed data quality assessments and provide these with the dataset (see *Technical Validation*). To reduce recruitment and retention biases we took steps including pseudo-stratification of recruitment (age and location), above-average study compensation, bonuses for continued participation, and continuous participant support during data-collection (see *Methods*). Despite these efforts, comparisons between our core sample and US-census data identified recruitment biases in the location, age, racial, ethnic, and political composition of the sample. Furthermore, we found retention biases with regards to age, education, race, and income (see *Technical Validation*).

For researchers wishing to apply post-stratification adjustments to the sample we calculated “raking” weights for several demographic variables. However, we note that due to the sparseness of the design and attrition over time, researchers interested in a particular research question in a specific subset of the data will likely need to conduct their own adjustments to counteract biases in their sample (e.g., selecting a stratified subsample or raking). As mentioned in the raking section, particular caution is recommended with respect to political and ideological beliefs. In the current sample conservative-leaning participants are underrepresented, yet political ideology has been shown to be a driving factor for COVID-related behaviors^78–80^. These issues limit the generalizability of the COVID-Dynamic data to a broader population. Yet, they have less impact on many analyses that focus on within-participant variation over time and relations between variables and psychological constructs.

Finally, as the declared goal of this study was to track psychological change across the pandemic, it is important to consider psychological biases in the sample. In accordance with previous research^81^, we found the low-completion rate group to be more anxious and depressed than the core sample. Additionally, the excluded, and low-completion rate groups were less conscientious and agreeable, and more neurotic than the core sample. Further, the excluded group showed less openness to experience, while the low-completion rate group showed more extraversion than the core group (Table 9). However, it is noteworthy that, relative to the community-based norms, the NEO personality factors^26^, trait anxiety (STAI^35^) and depression index (BDI-II^13^) averages for all groups (attrition and core) were within the normal range. By providing this detailed characterization of our sample, we aim to constrain interpretation as well as to situate our study in context and thereby provide a basis on which to integrate these data with other valuable COVID-19-related psychological datasets (e.g. the APS Global Collaboration on COVID-19^82^).

We strongly encourage researchers using this data to take these factors into consideration during data analysis and interpretation.

## Data Availability

All questionnaires were implemented in Qualtrics, tasks were implemented using the JsPsych toolbox^83^ (Version 6.3.1 and 6.0.5). Data processing, analyses and visualizations were conducted in Python 3.7^84,85^ and R^63^. All Qualtrics qsf and pdf files and task code, as well as preprocessing and visualization code is publicly available via github (https://github.com/adolphslab/CVD-DYN_datarelease). Additional Public Resources a) Web-based data explorer: http://coviddynamicdash.caltech.edu/shiny/coviddash/ b) Summary of COVID-19 Psychological Studies: https://coviddynamic.caltech.edu/resources/other-covid-studies c) 2020 Timeline: https://coviddynamic.caltech.edu/resources/timeline-2020-world-events.
